# Classification and Management of Portal Vein Thrombosis in Cirrhotic Patients: A Narrative Review

**DOI:** 10.7759/cureus.65869

**Published:** 2024-07-31

**Authors:** Abdulwahed A Alotay

**Affiliations:** 1 Department of Internal Medicine, Imam Mohammad Ibn Saud Islamic University (IMSIU), Riyadh, SAU

**Keywords:** interventional radiology, hepatocellular carcinoma, liver transplantation, anticoagulation, emerging therapeutics, portal vein thrombosis

## Abstract

Portal vein thrombosis (PVT) poses significant therapeutic challenges due to its complex pathophysiology and diverse clinical presentations. Recent advancements have spurred the development of new therapeutic approaches to enhance treatment efficacy and safety. This review synthesized emerging therapies for PVT based on a comprehensive literature search across major databases such as PubMed, EMBASE, and Web of Science, among others, focusing on studies published in the last decade. Anticoagulation therapy, particularly with novel oral anticoagulants (NOACs), emerged as beneficial in personalized treatment regimens. Innovative surgical techniques and improved risk stratification methods were identified as crucial in the perioperative management of PVT. Additionally, advances in cell therapy and medical treatments for hepatocellular carcinoma in the context of PVT were explored. Promising outcomes were observed with modalities such as Yttrium 90 and liver transplantation combined with thrombectomy, particularly in complex PVT cases associated with hepatocellular carcinoma, albeit on a limited scale. The reviewed literature indicates a shift towards individualized treatment approaches for PVT, integrating novel anticoagulants, refined risk assessment tools, and tailored interventional strategies. While these emerging therapies show potential for enhanced efficacy and safety, further research is essential to validate findings across broader patient populations and establish standardized treatment protocols.

## Introduction and background

The splenic and superior mesenteric veins, which drain the spleen and small intestines, respectively, confluence to form the portal vein. A thrombus forming in the portal vein and intrahepatic portal branches is the hallmark of portal vein thrombosis (PVT), a vascular disorder that can impair hepatic portal blood flow and have potentially fatal consequences such as portal hypertension and portal cholangiopathy [1]. The incidence and prevalence of PVT in cirrhosis have been observed to range from 8.4% to 11.2% and almost 26% [1]. The complexities of the portal venous system and its vital role in systemic and hepatic circulation make PVT a challenging clinical condition to treat. It is frequently linked to liver cirrhosis, cancers, and prothrombotic conditions [2]. Unresolved acute PVT is the cause of chronic PVT. Patients with persistent PVT may experience collateral blood flow surrounding the blockage, causing blood to bypass the liver. This condition is referred to as portal cavernoma or cavernous metamorphosis.

Historically, thrombolytic medication, anticoagulation, and surgical procedures have been used as PVT treatments. The mainstay of treatment, anticoagulation, attempts to promote recanalization and inhibit thrombus expansion [3]. Thrombolysis is an excellent treatment for clot resolution, but it is not commonly utilized because of the high risk of bleeding, particularly in patients who have underlying liver disease. Because of their invasive nature and associated procedural risks, surgical procedures, such as thrombectomy or shunt operations, are only considered in the most severe instances [4-6]. Advantages include reduced thrombus extension, a decreased incidence of hepatic decompensation, a better rate of recanalization (37%-93%), and increased survival [2].

However, there has been a rise in interest recently in creating fresh approaches to treating PVT, with an emphasis on improving current therapies as well as investigating novel pharmacological agents [7-8]. The pathophysiological mechanisms underlying thrombosis have been better understood, leading to the development of targeted treatments with the potential to increase safety and efficacy [9].

The goal of these newly developed PVT therapies is to overcome the shortcomings of current therapies and offer alternatives to individuals who are not suitable candidates for established treatments [10-13]. There is hope that these breakthroughs could drastically change the management of PVT and improve results for patients suffering from this difficult condition, as research and clinical studies are still underway. The PVT therapeutic landscape is always changing, which emphasizes the value of ongoing research and development in the field of vascular hepatology [10-14].

This narrative review aims to comprehensively assess and synthesize the latest research on emerging therapeutics for PVT. This includes evaluating the efficacy and safety profiles of novel pharmacological agents and treatment strategies that have been developed in response to the challenges posed by traditional PVT management.

## Review

Eligibility criteria

Table 1 mentions the inclusion and exclusion criteria devised for our review.

**Table 1:** Inclusion and exclusion criteria devised for this review. RCT, randomized controlled trials; PVT, portal vein thrombosis

Criteria	Inclusion	Exclusion
Study design	Randomized controlled trials (RCTs), cohort studies, case-control studies, case series, observational studies, systematic reviews, meta-analyses	Posters, editorials, and opinion pieces
Participants	Studies involving patients diagnosed with portal vein thrombosis	Studies not addressing patients with portal vein thrombosis
Interventions	Studies evaluating emerging therapeutics and novel treatment strategies for PVT	Studies not assessing emerging or novel therapeutic interventions for PVT
Outcomes	Resolution of thrombosis, prevention of thrombus progression, recanalization rates, improvements in liver function tests, reduction in the incidence of variceal bleeding, survival rates, adverse events, and quality-of-life measures	Studies not reporting specific outcomes relevant to PVT management
Publication date	Studies published from 2014 onward	Studies published before 2014

Historically, patients with liver impairment were not good candidates for the use of newer oral ACs due to the risk of bleeding and unknown efficacy [3]. Therefore, studies published before 2014 were not considered for inclusion in this review to ensure that the analysis reflected the most current advancements and consensus in the field of PVT treatment. The rapid evolution of medical research, particularly in thrombosis and anticoagulation, has led to significant changes in clinical practice guidelines and treatment modalities. As the review's primary focus was on emerging therapeutics, capturing the latest developments and innovations post-2014 was imperative, which might not have been addressed or available in the earlier literature. Furthermore, the exclusion of older studies aimed to minimize the inclusion of data that could be rendered obsolete due to newer, more effective treatments, changes in clinical approaches, and technological advancements.

Database search protocol

The database search protocol for this review was conducted across eight databases: PubMed, EMBASE, Web of Science, Cochrane Library, Scopus, ClinicalTrials.gov, Ovid MEDLINE, and Google Scholar. The search strategy incorporated a combination of Medical Subject Headings (MeSH) terms and Boolean operators to refine and focus the results, as elucidated further through Table 2.

**Table 2:** Search strings utilized across the different databases. MeSH, Medical Subject Headings; PDAT, publication date; EMBASE, Excerpta Medica Database; exp, Explosion (searches for the term and all its synonyms); py, publication year; TS, topic search (searches for the terms in the title, abstract, and keywords); mp, multipurpose (searches for the term in various fields); EDAT, entry date

Database	Search string
PubMed	(("Portal Vein"[MeSH Terms] OR "portal vein"[All Fields]) AND ("Venous Thrombosis"[MeSH Terms] OR ("venous"[All Fields] AND "thrombosis"[All Fields]) OR "venous thrombosis"[All Fields] OR ("portal"[All Fields] AND "vein thrombosis"[All Fields]))) AND ("2014/01/01"[PDAT] : "3000"[PDAT])
EMBASE	'portal vein thrombosis'/exp OR 'portal vein thrombosis' AND ('anticoagulant therapy'/exp OR 'anticoagulant agent'/exp OR 'novel anticoagulant' OR 'direct oral anticoagulants' OR 'DOACs') AND (2014- 2024)/py
Web of Science	TS=(("Portal Vein Thrombosis" OR "PVT") AND ("Anticoagulation" OR "Anticoagulant Therapy" OR "Thrombolysis" OR "Thrombolytic Agents") AND ("Clinical Trial" OR "Cohort Study" OR "Case Series")) AND PY=(2014-2024)
Cochrane Library	#1 MeSH descriptor: [Portal Vein Thrombosis] explode all trees #2 MeSH descriptor: [Anticoagulants] explode all trees or 'direct acting oral anticoagulants':ti,ab,kw or 'novel oral anticoagulants':ti,ab,kw in Trials #3 (#1 AND #2) AND (Publication Year from 2014 to 2024) in Trials
Scopus	TITLE-ABS-KEY ( "portal vein thrombosis" AND ("anticoagulation therapy" OR "emerging treatment" OR "novel therapy" OR "interventional radiology" OR "endovascular" OR "surgical management") AND PUBYEAR > 2013 AND PUBYEAR < 2024
ClinicalTrials.gov	(Portal Vein Thrombosis[AllFields] AND ("2014/01/01"[PDAT]: "3000"[PDAT]) AND ("Interventional Studies"[Filter] OR "Observational Studies"[Filter]))
Ovid MEDLINE	(exp Portal Vein Thrombosis/ OR portal vein thrombosis.mp.) AND (exp Anticoagulants/ OR anticoagulants.mp. OR exp Thrombolytic Therapy/ OR thrombolytic therapy.mp.) AND (exp Clinical Trials/ OR clinical trial.mp. OR exp Cohort Studies/ OR cohort study.mp.) AND '2014'.mp. [EDAT]
Google Scholar	allintitle: "portal vein thrombosis" "anticoagulation" "clinical trials" "novel treatments" -filetype:pdf -filetype:doc -filetype:ppt since 2014

Results

A paradigm shift in therapeutic strategies away from traditional ACs was indicated by Priyanka et al. [15] meta-analysis on the use of non-vitamin K antagonist oral anticoagulants (NOACs) for treating acute PVT in patients with or without cirrhosis, as Table 3 makes clear. Additionally, the study revealed that NOACs had a similar safety and efficacy profile, indicating that they may be used as a regular treatment for PVT. The significance of incorporating heterogeneous patient groups in research was underscored, as it augments our comprehension of the impact of NOACs on various patient demographics. Moreover, Priyanka et al. [15] made a significant contribution to the identification of NOAC adverse event patterns and clinical risk factors, which is essential for patient management. Regardless of the presence of cirrhosis, the adverse effects associated with NOACs and standard ACs for the treatment of acute PVT are similar. These include bleeding events (major and small) and anticoagulation failure (propagation of thrombus or recurrence of PVT). To improve patient outcomes, they also drew attention to gaps in the literature and indicated the necessity for innovative methods to estimate PVT risk.

**Table 3:** Anticoagulant strategies and safety in PVT management. NOACs, novel oral anticoagulants; PVT, portal vein thrombosis; MPV, main portal vein

Study ID	Theme	Findings
Priyanka et al. [15]	Anticoagulant Use in PVT	NOACs are being considered for PVT treatment, indicating a shift in therapeutic strategies.
Wang et al. [16]		Anticoagulant therapy is identified as a significant factor in PVT risk post-surgery, highlighting the need for careful management.
Priyanka et al. [15]	Safety and Efficacy	NOACs present a comparable safety profile and efficacy, suggesting their potential as a standard PVT treatment.
Priyanka et al. [15]	Patient Population Demographics	The inclusion of diverse patient groups provides a broader understanding of NOACs' impact across different PVT populations.
Wang et al. [16]		A focus on cirrhotic patients’ post-surgery emphasizes the importance of tailored therapeutic approaches.
Priyanka et al. [15]	Clinical Risk Factors	The study's findings contribute to a deeper understanding of the pharmacological impact of NOACs on PVT.
Wang et al. [16]		The identification of specific clinical risk factors for PVT allows for more targeted prevention strategies.
Priyanka et al. [15]	Adverse Events	The adverse event profiles of NOACs inform clinicians of the risks associated with their use in PVT management.
Wang et al. [16]		Understanding the potential for adverse events related to anticoagulant therapy aids in optimizing patient safety.
Priyanka et al. [15]	Literature Gap and Novel Algorithms	Recognition of literature gaps suggests areas for future research and the potential for NOACs in PVT treatment.
Wang et al. [16]		Novel predictive algorithms for PVT risk could lead to improved patient outcomes and personalized care.
Priyanka et al. [15]	Outcome Measures	Outcomes related to anticoagulation failure and bleeding events are crucial for evaluating therapeutic efficacy.
Wang et al. [16]		The deployment of metrics to evaluate predictive models could inform future therapeutic directions.
Priyanka et al. [15]	Technological Advancement	The findings suggest an openness to adopting new therapeutic monitoring methods in clinical practice.
Wang et al. [16]		Machine learning applications in predicting PVT risk represent a significant technological advancement in clinical assessment.
Onda et al. [17]	Incidence and Risk Factors	Portal vein thrombosis was found in 14.3% of patients post-hepatectomy. Prolonged Pringle maneuver time (>75 minutes) was a significant risk factor.
	Treatment and Outcomes	A novel classification and treatment strategy with a 91% recovery rate. Thrombus resolution in 85% of patients treated with anticoagulation. 93% clearance in MPV, 80% in hilar, 92% in peripheral region. Urgent thrombectomy was effective in grade 3 thrombosis.
	Correlation with Operative Procedures	Incidence of PVT varied with the type of hepatectomy, suggesting a relation between surgical extent and PVT risk.

In cirrhotic patients, Wang et al. [16] evaluated the risk of PVT following splenectomy and cardiac devascularization. The top two risk factors for PVT are AC therapy and antiplatelet aggregation therapy, which are followed by D-D (dimer), CHOL (cholesterol), and Ca (calcium). Their study, which highlighted the value of individualized treatment strategies, concentrated on the post-surgery outcomes for cirrhotic patients. Furthermore, risk factor analysis (RFA) for PVT, a novel predictive approach for PVT risk in cirrhotic patients following splenectomy and cardiac devascularization, was promoted by Wang et al. [16]. then used the risk indicators that were identified to build a support vector machine (SVM) predictive model. which might result in more individualized treatment and better patient results.

The incidence, risk factors, and clinical results of PVT following hepatectomy were assessed by Onda et al. [17]. After hepatectomy, they discovered a 14.3% incidence of PVT in the patients, and a longer Pringle maneuver time was a major risk factor. The Pringle maneuver is a surgical technique used to control bleeding during liver surgery. It involves clamping the hepatoduodenal ligament to temporarily stop blood flow through the hepatic artery and portal vein, thereby reducing blood loss. This maneuver is typically performed for a limited duration, usually up to 20 minutes, to prevent liver ischemia. A unique approach to classification and treatment was suggested. Depending on the degree of blockage and the location of the thrombus, patients were divided into three groups. Anticoagulation treatment was not administered to patients with peripheral thrombosis or main grade 1. Anticoagulation therapy was administered to individuals with major grade 2 or heparin thrombosis. Individuals classified as grade 3 should have an immediate surgical thrombectomy. In a substantial portion of cases, there was thrombus clearance and a high rate of recovery. Additionally, they proposed a relationship between the type of hepatectomy and the risk of PVT, suggesting that the amount of surgery may have an impact on the propensity to develop PVT (Table 3).

The complexities in the classification and management of PVT during liver transplantation are summarized below. Bhangui et al. [18] identified that, at the time of their study, nine classification systems for PVT existed, yet none were specifically tailored for surgical decision-making during LT. They proposed a new classification that bifurcated the nontumoral PVT into non-complex and complex categories, which could significantly influence the choice of portal inflow reconstruction as either physiological or non-physiological based on whether it addresses the preexisting prehepatic PHT from a functional/hemodynamic standpoint. The prevalence of complex PVT in cirrhotic patients being considered for LT was relatively low, ranging from 0% to 2.2%. Physiological reconstruction was favored as it could resolve preexisting prehepatic portal hypertension (PHT), unlike nonphysiological reconstruction. Various options for portal inflow reconstruction were evaluated, with physiological options including RPA and large vein anastomosis, while nonphysiological options involved CPA, PVA, and MVT.

For peri-transplantation medical management, ACs and transjugular intrahepatic portosystemic shunt (TIPS) were found to prevent progression to complex PVT, and endoscopic variceal eradication and embolotherapy were used to manage PHT. Post-LT PVT prevention continued with AC therapy. An algorithm for reconstruction was proposed, based on the presence or absence of large spontaneous or surgical portosystemic shunts.

Glinka et al. [19] found that the incidence of PVT in pediatric Living Donor Liver Transplantation (LDLT) with hyper-reduced left lateral segment grafts (LLSGs) was 9.52%. They suggested that a biliary-first approach, which involves performing biliary reconstruction after the PV anastomosis and before removing the vascular clamp, allows for freely rotating the liver with less risk of PV occlusion and thrombosis, thereby reducing PVT risk compared to traditional techniques. Outcomes of this approach were promising, with a 100% patient and graft survival rate and a median follow-up of 26.4 months, although vascular complications occurred in 14.3% of patients (Table 4).

**Table 4:** Portal vein thrombosis in liver transplantation: classification, management, and outcomes. LT, liver transplantation; PVT, portal vein thrombosis; RPA, right portal anastomosis; CPA, cavoportal anastomosis; PVA, portal venous anastomosis; MVT, mesenteric venous thrombosis; TIPS, transjugular intrahepatic portosystemic shunt; LDLT, living donor liver transplantation; LLSGs, left lateral segment grafts

Study ID	Theme	Findings
Bhangui et al. [18]	Classification Systems	Nine classification systems exist, nonspecific to surgical decision-making during liver transplantation (LT).
	Proposed New Classification	New classification divides PVT into noncomplex (Yerdel grades 1-3) and complex (Yerdel grade 4 or Jamieson and Charco grades 3-4). Portal inflow reconstruction is classified as physiological or nonphysiological.
	Prevalence of Complex PVT	Ranges from 0% to 2.2% in cirrhotic patients at the time of LT.
	Physiological vs. Nonphysiological Reconstruction	Physiological reconstruction resolves preexisting prehepatic portal hypertension (PHT). Nonphysiological does not resolve PHT.
	Options for Portal Inflow Reconstruction	Physiological options include RPA, large left gastric vein anastomosis, and large pericholedochal varix anastomosis. Nonphysiological options include CPA, PVA, and MVT.
	Peri-Transplantation Medical Management	Anticoagulants and TIPS prevent progression to complex PVT. Treat PHT with endoscopic variceal eradication and embolotherapy. Post-LT PVT prevention with anticoagulation.
	Algorithm for Reconstruction	Proposes an algorithm based on the presence or absence of large spontaneous or surgical portosystemic shunts.
Glinka et al. [19]	Incidence and Risk Factors	The incidence of PVT is 9.52% in pediatric LDLT with hyper-reduced LLSGs. The biliary-first approach reduces PVT risk compared to traditional techniques.
	Outcomes	100% patient and graft survival rates, with a median follow-up of 26.4 months. Vascular complications occurred in 14.3% of patients.
	Specific Techniques or Approaches	The biliary-first approach was used in 21 pediatric patients with hyper-reduced LLSGs, resulting in a reduction in PVT incidence.

In patients with PVT who planned to proceed with LT, PVT was classified into four classes based on the Yerdel classification. Patients with grades 1 and 2 PVT can usually be managed with portal thromboendovenectomy (PTEV). Patients with grades 3 and 4 PVT need more extensive procedures to ensure adequate portal inflow to the graft which is not always available worldwide. Puri et al. [20] described liver transplantation with grade 4 PVT with portal vein thrombectomy and augmentation from the inferior mesenteric vein via a jump graft as a successful intervention despite traditional views of PVT as a contraindication. This advanced surgical technique led to immediate decompression of the inferior mesenteric vein (IMV), increased portal pressure and velocity, and smooth postoperative recovery with long-term excellent graft function. Postoperative complications were minimal and manageable (Table 5).

**Table 5:** Therapeutic interventions and outcomes for portal vein thrombosis in hepatocellular carcinoma. IMV, inferior mesenteric vein

Study Name	Theme	Findings Interpretation
Puri et al. [20]	Therapeutic Intervention	Liver transplantation with portal vein thrombectomy and augmentation of portal flow via a jump graft.
	Safety and Efficacy	Successful liver transplantation despite the traditional view of PVT as a contraindication; no immediate postoperative complications were reported.
	Outcomes	Immediate decompression of IMV, increased portal pressure and velocity, smooth postoperative recovery, and long-term excellent graft function.
	Predictors of Success	Not explicitly discussed, but a successful outcome implies effective surgical technique and patient selection.
	Surgical Techniques and Management	An advanced surgical technique involving portal vein thrombectomy and construction of a venous conduit between the IMV and the portal vein to augment portal flow.
	Postoperative Management and Complications	Postoperative serous collection treated with percutaneous drainage; otherwise, a smooth post-transplant course.
	Long-Term Efficacy	Excellent graft function and patient well-being at six months post-transplant indicate a favorable long-term outcome.

As elucidated in Table 6, the prevalence and clinical manifestation of PVT in individuals with liver cirrhosis were the main topics of study for Amitrano et al. [1]. PVT was found in 11.2% of the cirrhotic patients treated between January 1998 and December 2002, according to their report. Remarkably, 43% of these patients had no symptoms at all, whereas 57% had symptoms, mostly from intestinal infarction and gastrointestinal hemorrhage. Additionally, this study showed a strong correlation between PVT and the prothrombin gene mutation 20210, suggesting a genetic risk factor that may affect PVT incidence in cirrhotic people. The findings highlighted that while PVT is usually asymptomatic, cirrhotic patients usually have severe liver disease that frequently manifests with potentially fatal consequences.

**Table 6:** Comparative analysis of studies on PVT management in cirrhotic patients: focus on prevalence, treatment efficacy, and safety. PVT, portal vein thrombosis; OR, odds ratio; DOAC, direct oral anticoagulant

Study	Key focus	Population and study period	Main findings	Safety and complications	Conclusion
Amitrano et al. [1]	Portal vein thrombosis (PVT) in cirrhotic patients	Cirrhotic patients with/without PVT, Jan 1998 to Dec 2002	11.2% had PVT; 43% asymptomatic; 57% symptomatic with complications like gastrointestinal hemorrhage and intestinal infarction.	Prothrombin gene mutation 20210 significantly associated with PVT	PVT can be asymptomatic but often presents with life-threatening complications; advanced liver disease prevalent in cirrhotic patients with PVT.
Qi et al. [2]	Efficacy and safety of anticoagulation for PVT in cirrhotic patients	Systematic review and meta-analysis, various time frames	Anticoagulation led to a pooled recanalization rate of 66.6%; a significant increase in complete recanalization with anticoagulation (OR = 4.16)	Bleeding complications ranged from 0% to 18%, pooled rate of 3.3%; no lethal complications were reported.	Anticoagulation achieves high recanalization rates in cirrhotic patients with PVT; randomized controlled trials are needed for further verification.
Intagliata et al. [3]	Safety of direct oral anticoagulants (DOACs) vs. traditional anticoagulation in cirrhosis	39 cirrhosis patients receiving anticoagulation, over three years	Similar rates of bleeding between DOAC and traditional groups; no significant predictors of bleeding found	Comparable safety profiles between DOAC and traditional anticoagulants; no drug-induced liver injury reported.	DOACs show potential as safe alternatives to traditional anticoagulants in cirrhosis; larger studies are needed.

Qi et al.'s [2] comprehensive review and meta-analysis assessed the safety and effectiveness of AC medication in the treatment of PVT in patients with cirrhosis. With a pooled recanalization rate of 66.6%, they synthesized data from sixteen studies and discovered that AC medication significantly improved portal vein recanalization. Additionally, they saw a statistically significant rise in the percentage of total recanalization in anticoagulation-treated individuals when compared to non-treated patients. There were no reported fatal complications, and the safety profile was deemed satisfactory with a bleeding problems rate ranging from 0% to 18%. Despite their call for additional randomized controlled trials to corroborate these results and gain a better understanding of the risk-benefit ratio of anticoagulation in these patients, their conclusion emphasized the effectiveness of anticoagulation in obtaining high recanalization rates.

Direct oral anticoagulants (DOACs) and conventional anticoagulation techniques were examined for patient safety in cirrhosis patients by Intagliata et al. [3]. This three-year trial, which included 39 patients, did not find any appreciable differences in the incidence of bleeding between the groups treated with DOACs and those getting conventional anticoagulation. There was no evidence of drug-induced liver damage, and the safety profiles of the two anticoagulation techniques were equivalent. The study found that, although larger studies were suggested to further evaluate their safety and efficacy, DOACs may provide cirrhotic patients with a promising alternative to conventional ACs (Table 6).

Scheiner et al. [4] involved 122 patients with cirrhosis-associated non-malignant PVT (Table 7). They investigated the effects of long-term AC medication while keeping an eye on ascites, albumin levels, and liver enzymes (AST/ALT). According to the findings, anticoagulation was linked to noticeably greater rates of PVT regression; this association was especially strong in patients who had already had decompensation before the diagnosis of PVT. Significant bleeding episodes were not linked to traditional anticoagulation in this investigation, while one bleeding incident was related to the use of direct oral anticoagulant (DOAC) therapy. According to the study's findings, anticoagulation is a safe and efficient treatment for nonmalignant PVT in cirrhosis, with possible advantages for improved liver function and decreased hepatic damage.

**Table 7:** Overview of interventional strategies and outcomes in the management of PVT across different patient populations. AC, anticoagulation; AST, aspartate aminotransferase; ALT, alanine aminotransferase; DOAC, direct oral anticoagulant; TIPS, transjugular intrahepatic portosystemic shunt; JAK2, Janus kinase 2; PVT, portal vein thrombosis

Study	Population and context	Intervention and methods	Key findings and outcomes	Conclusions
Scheiner et al. [4]	122 patients with nonmalignant PVT in cirrhosis	Long-term anticoagulation (AC) therapy, comparing outcomes with/without AC. Parameters like AST/ALT, ascites, and albumin levels were monitored.	AC was associated with higher PVT regression rates (58% with AC vs. 28% without; significant in decompensated before PVT diagnosis subgroup). No significant AC-associated bleeding events, one bleeding event with DOAC treatment.	Supports the use of AC in nonmalignant PVT in cirrhosis, indicating safety and effectiveness, particularly in improving liver functions and reducing hepatic injury.
Chamarthy et al. [5]	Patients with symptomatic portomesenteric venous thrombosis	Various catheter-based interventions such as TIPS and percutaneous transhepatic approaches for acute and subacute PVT.	Different approaches have specific indications, advantages, and disadvantages, emphasizing the importance of selecting the right technique based on patient-specific factors.	Catheter-based interventions are safe and effective for PVT treatment, especially in cases refractory to medical therapy. Early diagnosis and tailored treatment are critical.
Klinger et al. [6]	17 patients with acute noncirrhotic, nonmalignant PVT	Transjugular interventional therapy, including thrombectomy, local fibrinolysis, and potentially TIPS depending on thrombus resolution.	High recanalization success (94.1%), with good secondary patency rates. Some complications like heparin-induced thrombocytopenia occurred. JAK2 mutation was a predictor of technical success.	Transjugular interventional therapy is recommended for acute non-cirrhotic PVT, particularly effective in preventing serious complications like bowel infarction.

To treat symptomatic portomesenteric venous thrombosis, especially in cases that are not responding to medication, Chamarthy et al. [5] examined several catheter-based procedures. Techniques including the TIPS and percutaneous transhepatic methods were covered in detail in the review. The pros, disadvantages, and particular indications of each approach were highlighted, emphasizing the significance of customizing the intervention to the unique situations of each patient as well as anatomical concerns. The study emphasized the need for early identification and timely, tailored therapy to prevent long-term problems, while also highlighting the efficacy and safety of catheter-based therapies.

In 17 patients with acute noncirrhotic, nonmalignant PVT, Klinger et al. [6] investigated the use of transjugular interventional therapy. The primary justification for intervention was an impending danger of intestinal infarction. Transjugular thrombectomy and local fibrinolysis were part of the treatment plan; depending on how well the thrombus resolved, TIPS might also be added. Recanalization was highly successful with the therapy, and secondary patency rates were maintained. Technical success was found to be predicted by the existence of the JAK2 V617F mutation both in the short and long term, despite the observation of certain problems, such as heparin-induced thrombocytopenia. Transjugular interventional therapy was suggested by this study as a useful treatment for acute noncirrhotic PVT, especially in cases where there is a significant risk of serious consequences such as bowel infarction (Table 7).

A study of the literature on noncirrhotic PVT patients who did not react to AC medication was done by Cheng et al. [7]. They examined information from trials that had 134 patients and were carried out after 2000. The rates of radiological recanalization and clinical improvement following thrombolysis were the main outcomes that were assessed. According to the study, thrombolysis produced an 84% recanalization rate and an 86% reduction in symptoms. Relatively few problems occurred; just 7% of significant complications were recorded. The study found that when anticoagulation fails, thrombolysis is a safe and effective treatment option for PVT in non-cirrhotic patients. However, it also recommended the development of standardized protocols to assess secondary outcomes and the efficacy of various thrombolysis methods.

Lv et al. [8] concentrated on patients with nonmalignant PVT who were cirrhotic. To assess an individualized therapy regimen comprising a wait-and-see strategy, anticoagulation, TIPS (transjugular intrahepatic portosystemic shunt), and a combination of TIPS and anticoagulation, they used a prospective research design with 396 consecutive patients. The degree of the thrombus and the stage of liver disease were the main factors influencing treatment choices. 81.3% of patients had partial or total recanalization, according to the study. However, the study also found that serious side effects like hepatic encephalopathy and severe bleeding were common. The results showed that anticoagulation-especially with drugs other than warfarin and TIPS was linked to better survival and higher rates of recanalization, even if there was a significant risk of severe bleeding, particularly in patients with full SMV thrombosis.

In their study of the prevalence, clinical manifestation, and treatment of PVT in patients with cirrhosis, Gadani et al. [9] suggested a unique protocol for management based on a range of clinical situations. To better understand the intricate nature of PVT management, their study included expert talks in addition to a survey of the literature. The evaluation emphasized that, unless there is a contraindication due to a disease such as bleeding varices or mesenteric ischemia, anticoagulation should be the first option of treatment for one-third of PVT patients in cirrhosis. Along with TIPS installation, the study also evaluated the possible advantages of minimally invasive procedures such as chemical thrombolysis and mechanical thrombectomy. The findings indicated that more research was necessary to identify the best ACs as well as the best times and strategies for minimally invasive therapies in particular situations (Table 8).

**Table 8:** Efficacy and safety of various management strategies for portal vein thrombosis (PVT) in cirrhotic and noncirrhotic patients. TIPS, transjugular intrahepatic portosystemic shunt

Study	Patient Group	Intervention	Methods	Outcome	Conclusion
Cheng et al. [7]	Noncirrhotic patients with PVT do not respond to anticoagulation.	Thrombolysis	Literature review of studies post-2000, involving 134 patients	84% recanalization; 86% symptom improvement; 7% major complications	Thrombolysis is deemed effective and safe for non-cirrhotic PVT when anticoagulation fails. Calls for standardized future studies to explore thrombolysis techniques and protocols.
Lv et al. [8]	Cirrhotic patients with nonmalignant PVT	Individualized management: Wait-and-see, anticoagulation, TIPS, TIPS + anticoagulation	A prospective study involving 396 patients, treatment based on liver disease stage and thrombus extent	81.3% achieved recanalization; significant rates of major bleeding and hepatic encephalopathy	TIPS and anticoagulation (especially non-warfarin) are associated with better outcomes. Complete superior mesenteric vein thrombosis is linked to poorer outcomes.
Gadani et al. [9]	Cirrhotic patients with PVT	Review of medical and interventional radiology management	Literature review and expert discussions	Varied outcomes based on clinical scenarios and PVT extent	Emphasizes complex management of PVT, highlighting anticoagulation as the initial treatment. Recommends ongoing research into minimally invasive treatments and anticoagulation strategies.

A retrospective analysis was carried out by Luca et al. [10] on seventy cirrhotic patients with non-tumoral PVT who had TIPS placement at a tertiary-care center between January 2003 and February 2010 (Table 9). According to the study, TIPS placement was 100% successful and came with no significant issues. After the treatment, 57% of the patients showed complete recanalization of the portal venous system, and 30% had a significant decrease in thrombosis. For the rest, though, there was no improvement. Additionally, patients with bare stents had higher TIPS malfunction rates (38% at 12 months and 85% at 24 months) than patients with covered stents (21% at 12 months and 29% at 24 months), according to the study. Within 24 months, 27% to 32% of the individuals experienced encephalopathy. The survival rates were good at 1, 12, and 24 months - 99%, 89%, and 81%, respectively, despite these difficulties. The study's conclusion, which suggests more research to establish TIPS as the best course of treatment for this patient population, is that TIPS installation gives good long-term outcomes for controlling non-tumoral PVT in cirrhotic patients.

**Table 9:** Effectiveness of TIPS and PVR-TIPS in managing portal vein thrombosis (PVT) in cirrhotic patients: outcomes and long-term survival rates. TIPS, transjugular intrahepatic portosystemic shunt; PVR-TIPS, partially covered transjugular intrahepatic portosystemic shunt

Study	Patient group	Intervention	Methods	Outcome	Conclusion
Luca et al. [10]	Cirrhotic patients with non-tumoral PVT	TIPS placement	Retrospective review of 70 patients treated from 2003 to 2010	Successful TIPS placement in all; 57% complete recanalization; 30% marked decrease in thrombosis; TIPS dysfunction rates higher in bare stents; 27%-32% developed encephalopathy	TIPS showed promising long-term outcomes for non-tumoral PVT in cirrhotic patients. Recommends further research to confirm TIPS as the best treatment option for this condition.
Thornburg et al. [11]	Cirrhotic patients with PVT, potential liver transplant candidates	PVR-TIPS	Retrospective analysis of 61 patients, median follow-up of 19.2 months	Technical success in 98%; 92% maintained patency; 8% had recurrent thrombosis; 39% underwent transplantation with a high success rate; 82% five-year survival rate	PVR-TIPS is safe, effective, and durable for managing chronic PVT in cirrhotic patients awaiting liver transplantation.

Sixty-one cirrhotic individuals with PVT who might benefit from liver transplantation were examined by Thornburg et al. [11]. PVR-TIPS was performed on these patients to improve their transplantability. Over a median follow-up period of 19.2 months, the surgery maintained a PV/TIPS patency rate of 92% and achieved a technical success rate of 98%. Eight percent of the patients had recurrent thrombosis, with those who first presented with full PVT being particularly affected. Transient encephalopathy and TIPS stenosis, which affected 22% and 18% of the patients, respectively, were the most frequent adverse effects. Remarkably, 96% of the transplants performed on 39% of the patients eventually were effective. A noteworthy five-year overall survival rate of 82% was also discovered by the study. The results supported the use of PVR-TIPS to enhance transplantation outcomes by highlighting its status as a long-lasting, safe, and efficacious strategy for cirrhotic patients with chronic PVT awaiting liver transplantation (Table 9).

Discussion

The corpus of included papers within this review [15-23] offers a panoramic view of the evolving landscape of PVT therapeutics, each contributing unique insights pertinent to the overarching objective of our review, which was to delineate the advancements in the treatment of PVT. Priyanka et al. [15] and Wang et al. [16] both focused on anticoagulation therapy in the context of PVT, with the former discussing NOACs and the latter examining AC management post-surgery in cirrhotic patients. The common ground between these studies was the proposal of individualized treatment regimens and the development of predictive algorithms for PVT risk, signifying a shared emphasis on personalized medicine. However, the divergence in their findings was marked by Priyanka et al.'s [15] broader examination of NOACs across various patient demographics, while Wang et al. [16] honed in on the post-surgical subset of cirrhotic patients.

Onda et al. [17] introduced a novel classification and treatment strategy predicated on their finding of the Pringle maneuver time as a significant risk factor for PVT, paving the way for a surgical approach to PVT that is distinct from the medically-oriented interventions discussed in the aforementioned studies.

The surgical lens provided by Bhangui et al. [18] and Glinka et al. [19] offered another dimension to the collective understanding of PVT management. Bhangui et al. [18] proposed a new classification system for non-tumoral PVT specifically tailored to surgical decision-making during liver transplantation. Glinka et al. [19], on the other hand, advocated for a *biliary-first *approach in pediatric LDLT, reporting a reduction in PVT risk. Both studies proffered surgical innovation as a key to improved outcomes, sharing a commonality in their focus on operative strategies, albeit in different patient populations.

The study by Xu et al. [21] presented findings that align with those observed by Priyanka et al. [15] in our analysis, both of which highlighted the efficacy of NOACs in the management of PVT in cirrhotic patients. Xu et al. [21] conducted a meta-analysis that demonstrated a higher recanalization rate with NOACs compared to traditional ACs without an increased risk of bleeding. This complements Priyanka et al.'s [15] broader examination of NOACs across patient demographics and reinforces the potential of NOACs as a superior treatment option for PVT recanalization.

Wu et al. [22] echoed the individualized approach to PVT management that was a central theme in our analysis. Their work supported the notion that treatment should be tailored based on the thrombosis's size, extent, the presence of symptoms, comorbidities, and whether the PVT is acute or chronic. Although Wu et al. [22] discussed the use of low molecular weight heparin and vitamin K antagonists (VKAs), they acknowledged the emerging evidence supporting the use of direct-acting oral ACs, which is consistent with the findings of Xu et al. [24] and our study's findings regarding the efficacy of NOACs.

Nicoară-Farcău et al. [23], similar to Wang et al. [16] from our analysis, underlined the necessity for a better understanding of the pathophysiology and risk factors of PVT in cirrhotic patients. They stressed the need for a homogeneous staging of PVT to facilitate collaborative studies. This perspective resonates with the emphasis on predictive algorithms for assessing PVT risk highlighted by Priyanka et al. [15] and Wang et al. [16]. However, Nicoară-Farcău et al. [23] also pointed out the paucity of evidence supporting current treatment algorithms, suggesting that the consensus is largely based on expert opinion. This indicates a gap in evidence-based treatment protocols, which both our analysis and the studies by Xu et al. [24] and Wu et al. [22] aim to address by providing more concrete data on the effectiveness and safety of various anticoagulation strategies.

**Figure 1:**
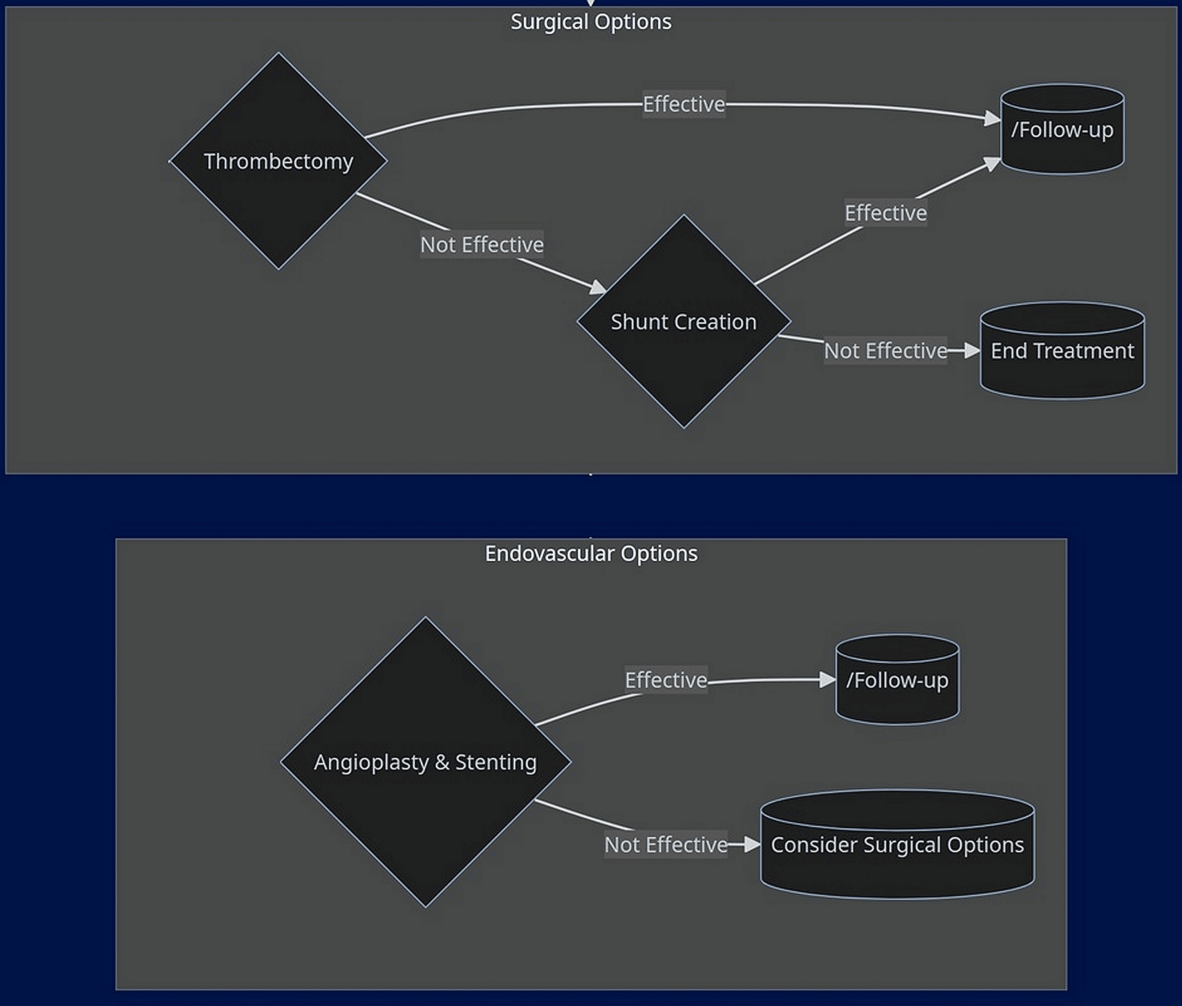
Invasive treatment approaches for PVT. PVT, portal vein thrombosis Image created by the authors using Mermaid Diagramming and Charting Tool (https://mermaid.js.org/).

The treatment of PVT is multifaceted, with the initial approach often starting with anticoagulation therapy (Figure 1).

This regimen typically begins with evaluating the suitability of DOACs for the patient [14]. If DOACs are effective and well-tolerated, the patient may continue with long-term maintenance therapy. However, if DOACs are not tolerated or contraindicated, VKAs or low-molecular-weight heparin (LMWH) may be considered as alternatives.

For cases where anticoagulation and thrombolysis are not viable or sufficient, surgical options such as thrombectomy and shunt creation are considered. The decision to proceed with surgery depends on the effectiveness of thrombectomy; if it is not effective, shunt creation may be the next step [24-25]. Endovascular options, including angioplasty and stenting, offer less invasive alternatives to surgery. The success of these procedures is closely monitored, and if they fail to yield the desired results, surgical options may be revisited. Each step in the treatment algorithm is carefully considered, with follow-ups ensuring that the chosen modality provides the best possible outcome for the patient [26].

**Figure 2:**
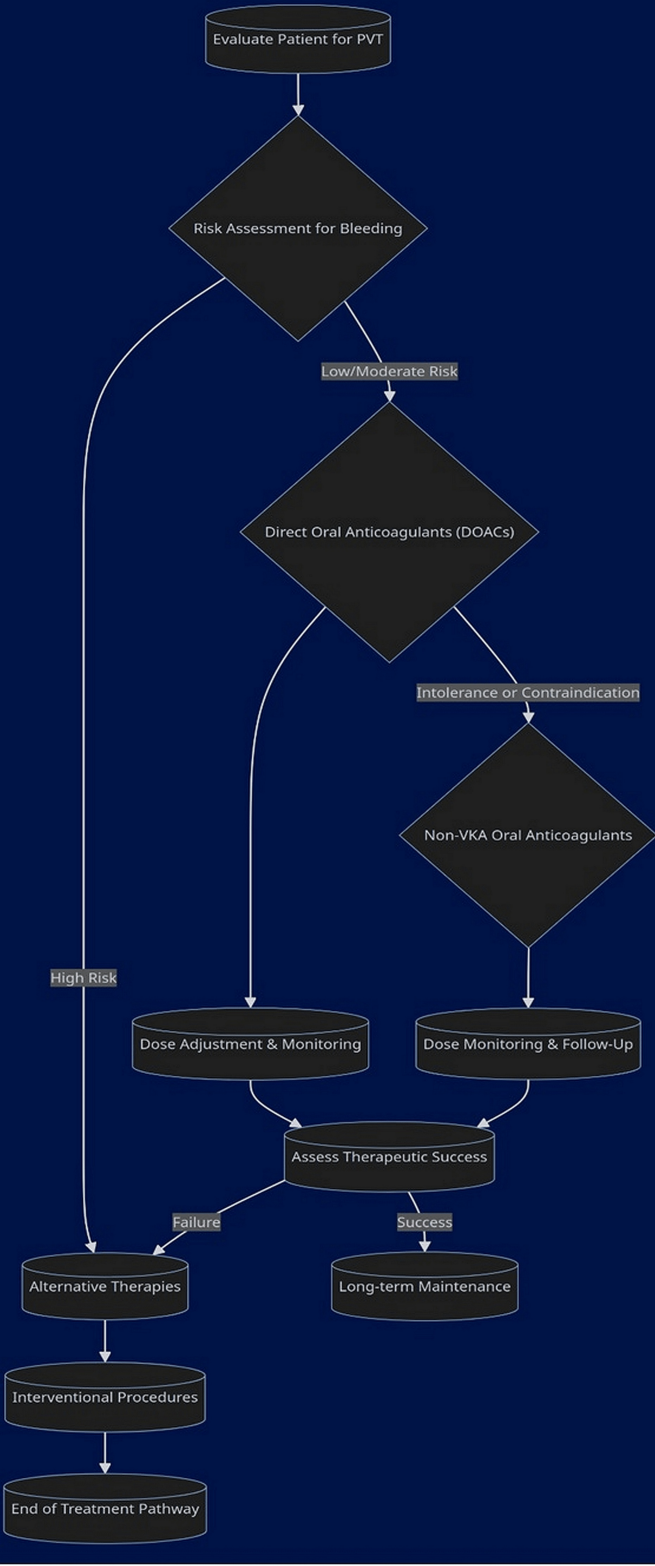
Advanced anticoagulation strategies for PVT. PVT, portal vein thrombosis; VKA, vitamin K antagonist Image created by the authors using Mermaid Diagramming and Charting Tool (https://mermaid.js.org/).

Patients are initially assessed for bleeding risk, which informs the choice between DOACs and alternative therapies (Figure 2).

Dose adjustment and close monitoring follow the initiation of DOACs, while intolerance or contraindication to DOACs leads to the consideration of non-VKA oral ACs. Interventional procedures serve as a secondary pathway for cases where anticoagulation poses too great a risk or is ineffective.

Thrombosis development follows the principles of Virchow's triad, which includes the coexistence of venous stasis, a state prone to coagulation, and damage to the vascular lining [11]. These elements are interconnected and can collectively contribute to the onset of PVT [12]. Studies have observed that post-hepatectomy, there is a notable increase in coagulation potential, as evidenced by decreased serum prothrombin (PT) activity levels on the first postoperative day (POD 1) among patients who develop PVT [13]. The Pringle maneuver, while effective in managing hemorrhage from the portal vein during liver resection [14], can inadvertently induce venous stasis and damage to the endothelium [12]. A prolonged application of this technique has been identified as a substantial risk factor for PVT, corroborating earlier findings [3].

**Figure 3:**
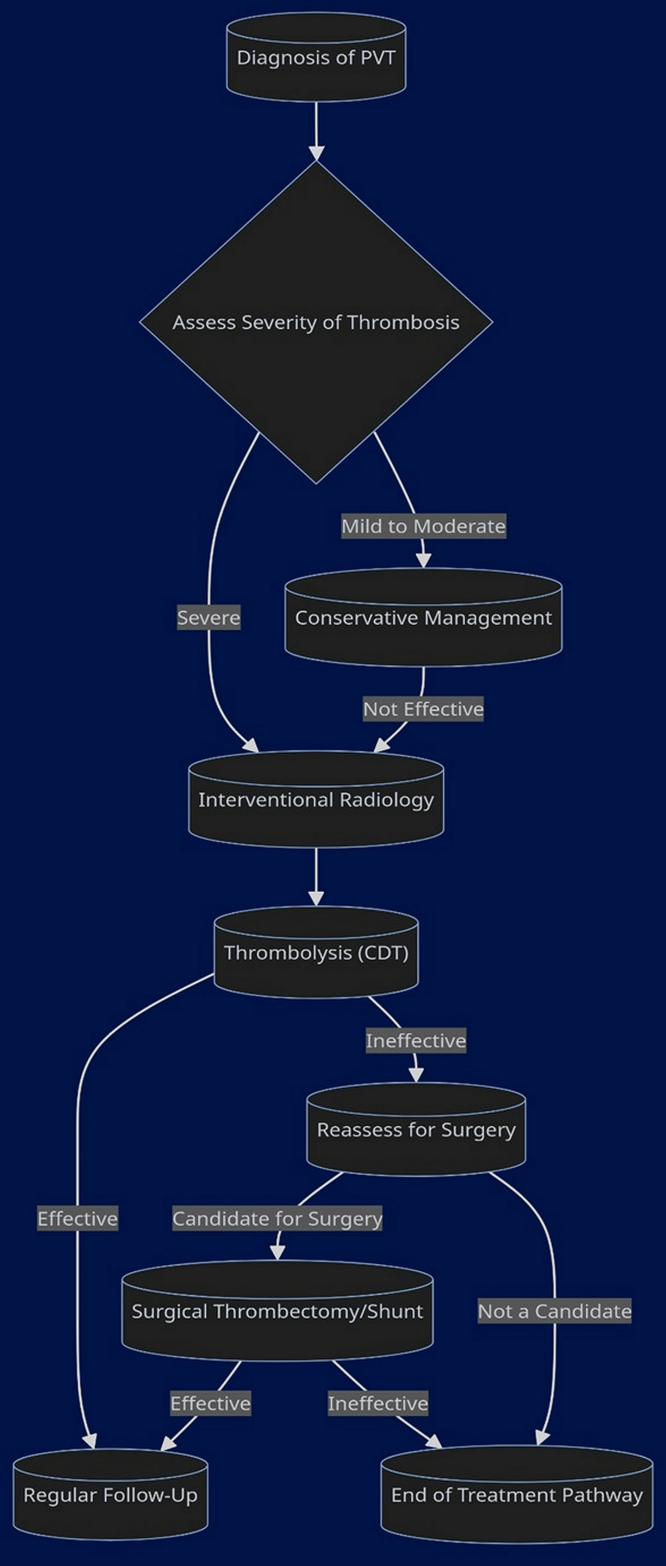
Interventional and surgical therapies for PVT. PVT, portal vein thrombosis Image created by the authors using Mermaid Diagramming and Charting Tool (https://mermaid.js.org/).

The effectiveness of AC therapy in the context of PVT has been evaluated predominantly through retrospective cohort studies due to a scarcity of randomized controlled trials comparing these interventions with a placebo. The reported recanalization rates with conventional ACs, including VKAs and LMWH, vary, with any level of recanalization achieved in 39% to 75% of cases [27-32], and complete recanalization in 33 to 45% [33]. The timing of anticoagulation commencement post-thrombus formation is a critical factor, with improved outcomes when therapy begins within 14 days according to some studies [34], or within six months in others [35]. Other parameters linked to favorable anticoagulation responses include less severe liver disease [36-39], less extensive thrombosis [40-41], superior mesenteric vein (SMV) occlusion under 50% [42], lower platelet counts [43], absence of prior bleeding due to PHT [41], and smaller initial spleen size [42]. The response to anticoagulation therapy does not appear to be significantly influenced by the presence of a thrombophilic disorder or the ratio of factor VIII to protein C [43-44]. Long-term anticoagulation using enoxaparin or rivaroxaban rather than warfarin was associated with a decreased risk of re-thrombosis and improved survival, without increasing the risk of bleeding. However, the presence of complete SMV thrombosis was associated with a lower recanalization rate, increased risk of major bleeding, and poor prognosis [8] (Figure 3).

Following the diagnosis, the severity of thrombosis is assessed to determine the appropriate treatment route [41]. Severe cases may undergo thrombolysis via catheter-directed techniques (CDT), with successful treatment leading to regular follow-up. Ineffective thrombolysis prompts reassessment for surgical options, such as thrombectomy or shunt creation. Mild-to-moderate cases may be managed conservatively, with a shift to interventional strategies if conservative management is not effective [39]. Direct or indirect thrombolysis approaches of the PVT offer the ability to treat the thrombus and restore portal vein patency. The TIPS approach is a safe and effective technique for direct portal vein access and treatment of PVT with the added advantages of decreased bleeding complications, and the ability to address portal hypertension and variceal bleeding at the same time [5].

Patients with cirrhosis and PVT should be offered anticoagulation as a first-line therapy if not contraindicated. TIPS placement provides an alternative option for recanalization in the presence of contraindications for anticoagulation or case of refractory PVT [9]. TIPS placement is frequently used in patients with PVT and cirrhosis after mechanical thrombectomy and/or chemical thrombolysis, which allows for a reduction of the pressure in the main portal vein and provides outflow for the blood without stagnation [10].

Limitations

The study, while comprehensive in its scope, faced several limitations that must be acknowledged to contextualize the findings. One significant limitation was the heterogeneity of the study designs included in the review. The varying methodologies, ranging from retrospective analyses to clinical trials, could have introduced a degree of bias and affected the comparability of outcomes, thereby potentially limiting the generalizability of the findings. Another limitation was the potential for publication bias, as studies with positive outcomes are more likely to be published than those with negative or inconclusive results. This could have resulted in an overrepresentation of studies demonstrating the efficacy of certain therapeutic interventions for PVT, skewing the overall conclusions.

Furthermore, the studies included in the review predominantly focused on short-term outcomes, providing limited insight into the long-term efficacy and safety of the emerging therapeutics for PVT. The chronic nature of PVT and its management necessitates long-term data to fully understand the implications of these treatments over an extended period. The specific patient populations studied also presented a limitation. For instance, certain studies concentrated on patients with specific underlying conditions, such as HCC, which may not be representative of the broader population of patients with PVT. This limits the applicability of the findings to all patients suffering from PVT.

Additionally, the impact of emerging therapeutics on quality of life and patient-reported outcomes was not adequately addressed in the reviewed studies. These factors are crucial for evaluating the true benefit of any intervention and should be considered in future research efforts. Furthermore, the rapid pace of medical advancements means that the literature review may not have captured the most cutting-edge therapies being developed or tested, which could influence the current understanding and management of PVT.

Cardiological recommendations

The synthesis of the information from this review suggests several recommendations for clinical practice. First and foremost, there is a strong recommendation for the adoption of individualized treatment regimens for patients with PVT, taking into account the unique clinical scenarios and patient demographics. This approach should be informed by the continued development and implementation of predictive algorithms designed to assess the risk of PVT, thereby enabling more personalized and effective anticoagulation therapies.

In light of the findings regarding the use of NOACs, it is recommended that these drugs be considered as a viable option for anticoagulation therapy in PVT management, with the caveat that careful patient selection and monitoring are crucial due to the diversity of patient responses. For patients undergoing surgical interventions, particularly those with cirrhosis, AC management should be carefully tailored to mitigate postoperative PVT risk.

For surgical approaches to PVT, including liver transplantation, it is recommended that novel classification systems and treatment strategies, such as those suggested by recent studies, be integrated into decision-making processes to optimize outcomes. This includes considering factors such as the Pringle maneuver time and adopting new surgical techniques that may reduce the risk of PVT.

The therapeutic landscape for patients with hepatocellular carcinoma (HCC) and concurrent PVT is recommended to be expanded to consider the safety and efficacy of various treatment modalities that do not necessarily compromise due to the presence of PVT. Treatments such as radioembolization and sorafenib should be evaluated on an individual basis to determine the best course of action for each patient.

For pediatric patients, particularly those with underlying metabolic disorders, the exploration of cell therapy options should be approached with caution. A thorough evaluation of the safety and potential thrombotic complications associated with such therapies is recommended before their widespread adoption.

In cases of unresectable HCC with PVT, nonsurgical interventions such as Yttrium 90 therapy should be considered as part of the treatment arsenal, with recognition of their utility in managing complex cases. Conversely, in situations where liver transplantation is feasible, the integration of portal vein thrombectomy can be beneficial and should be considered when planning surgical intervention strategies.

## Conclusions

The collective findings from the reviewed studies underscored a paradigm shift in the approach toward PVT management, with a discernible trend toward personalized medicine. The investigations revealed that anticoagulation therapy, particularly with NOACs, was effective across a range of patient populations, though the efficacy was contingent upon patient-specific factors, which necessitated individualized treatment protocols. Studies focusing on post-surgical management in cirrhotic patients further corroborated the value of tailoring anticoagulant therapy to individual clinical scenarios. Surgical interventions for PVT, particularly in the setting of liver transplantation, also reflected an evolution in practice. The introduction of novel classification systems and treatment strategies, informed by risk factors such as the duration of the Pringle maneuver, suggested a refinement in surgical decision-making aimed at minimizing PVT risk and optimizing patient outcomes.

In the realm of HCC, the presence of PVT did not significantly detract from the efficacy of certain treatments, such as radioembolization and sorafenib, indicating that PVT should not preclude the use of these therapies in affected patients. These findings suggest that the management of HCC in the context of PVT can be versatile and should be customized based on the patient's overall clinical presentation. Pediatric care presented a unique set of considerations, particularly with the introduction of cell therapies for metabolic disorders, which exhibited potential thrombotic complications. This highlighted the need for meticulous safety assessments and monitoring protocols when considering such treatments for younger patients.
